# Emergency surgery without stabilization prior to surgical repair for total anomalous pulmonary venous connection reduces duration of mechanical ventilation without reducing survival

**DOI:** 10.1186/s13019-021-01559-y

**Published:** 2021-08-02

**Authors:** Linyun Xi, Chun Wu, Zhengxia Pan, Ming Xiang

**Affiliations:** 1 Department of Cardiothoracic Surgery, Ministry of Education Key Laboratory of Child Development and Disorders, National Clinical Research Center for Child Health and Disorders, China International Science and Technology Cooperation base of Child Development and Critical Disorders, Children’s Hospital of Chongqing Medical University, No.136, Zhongshan 2nd Road, Yuzhong Dis, Chongqing, 400014 China; 2 Chongqing Key Laboratory of Pediatrics, No.136, Zhongshan 2nd Road, Yuzhong Dis, Chongqing, 400014 China

**Keywords:** Total anomalous pulmonary venous connection, Surgery, Outcome

## Abstract

**Background:**

To examine two different operation timing for treating patients with a total anomalous pulmonary venous connection (TAPVC) who need emergency surgery and to summarize the effects of the two operation strategies.

**Methods:**

A retrospective review of 54 patients with TAPVC who underwent operations within 72 h of presentation between December 2010 and July 2019 at a single institution was conducted. All patients exhibited respiratory or hemodynamic instability that required mechanical ventilation and inotropic support. Forty-four patients received emergency operations between 24 to 72 h due to stabilization of the patient’s condition. Stable hemodynamics were achieved, and a stable internal milieu was maintained before the operation. These patients comprised the Stable group (SG). Rather than being subjected to efforts to obtain stable hemodynamics and maintain a stable internal milieu, ten patients received emergency operations immediately within 24 h of diagnosis or an emergency operation is performed immediately due to uncorrectable acidosis or progressive cardiovascular collapse. These patients comprised the Unstable group (UG). The hospital course, operative data, and outpatient records were reviewed.

**Results:**

In SG group, there were 23 exhibited the supracardiac type, 15 exhibited the cardiac type, 4 exhibited the cardiac type, and 2 exhibited the mixed cardiac type,3 patients were premature, the rest was term infant, PDA was the most common comorbidities (28 patients), the next is severe tricuspid valve regurgitation (21 patients). In UG group, there were 3 exhibited the supracardiac type, 4 exhibited the cardiac type, 3 exhibited the cardiac type, and no patient exhibited the mixed cardiac type, only 1 patient was premature, the rest were term infant. PDA (6 patients) and severe tricuspid valve regurgitation (5 patients) were the top two comorbidities. The median weight, median age at surgery, mean cardiopulmonary bypass (CPB) duration and mean aortic cross-clamp (ACC) duration were not significantly different between the two groups. The median postoperation durations of ventilator support were 8.1 ± 4.6 (2–13) days in the SG group and 4.9 ± 2.1 (2–18) days in the UG group, resulting in a significant difference (*p* = 0.008), the Post-op days in ICU and Days of hospitalization were 8.64 ± 4.04 days and 19.9 ± 4.27 days in the SG group and 5.6 ± 2.01 days and 14.7 ± 1.75 days in the UG group (*P* = 0.026 and 0.002). There were 12 hospital mortalities (27.3%) in the SG group and 2 hospital mortalities (20%) in the UG group, resulting in no significant difference in mortality (*p* = 0.636). Postoperative complications, such as low cardiac output and arrhythmia, were not significantly different between the two groups. The survival rates in the UG and SG groups at 5 years were 87.5 and 89.9%, respectively. There was no difference in survival between the two groups at the latest follow-up (SG group 89.9% versus UG group 87.5%, *p* = 0.8115).

**Conclusion:**

An emergency operation should be performed immediately without any delay, it can reduce duration of mechanical ventilation and Days of hospitalization without reducing mortality.

## Introduction

The outcomes of the surgical repair of a total anomalous pulmonary venous connection (TAPVC) have historically improved, with the mortality rates reported in the literature ranging from 10 to 20% [1, 2] or even below 7% [3, 4], while emergency surgery reported had mortality rates as high as 25% [1, 2].some patients have a risk of progressive pulmonary hypertension (PH) due to pulmonary venous obstruction (PVO), and other patients present with increasing cyanosis, tachypnea, hepatomegaly, hypoxia, gasping, or poor feeding [5] due to a delayed diagnosis. All these patients have a risk of progressive PH, suffer from severe respiratory distress and metabolic acidosis and require immediate intubation and inotropic support; more importantly, an emergency operation is needed. However, there are two viewpoints pertaining to the encounter of this situation. In some patients, the specialist would make efforts to obtain stable hemodynamics, maintain a stable internal milieu and then perform an emergency operation. This process can take several days. In other patients, an emergency operation is performed immediately instead of trying to obtain stable hemodynamics and maintain a stable internal milieu, what’s more, preoperative medical stabilization is impossible in some cases, even with unstable hemodynamics, an emergency operation is performed. Because of the rarity of reports on these patients who must undergo immediate emergency operations, we conducted a retrospective study to assess the outcomes of such patients. We aimed to examine two different operation timing for treating patients with a total anomalous pulmonary venous connection (TAPVC) who need emergency surgery and to summarize the effects of the two operation strategies.

## Material and methods

### Record review and definitions

In this retrospective analysis, we reviewed the clinical records of patients with isolated TAPVC between December 2010 and July 2019. Patients with single ventricles, atrial isomerism and other complex congenital heart diseases or other complex comorbidities were excluded. Patient characteristics, such as weight and age, pulmonary venous anatomy, demographics, preoperative data, operative data, postoperative data and follow-up data, were recorded. A total of 234 patients who were diagnosed with TAPVC underwent surgical repair at the Children’s Hospital of Chongqing Medical University. Fifty-four patients (23.1%) needed emergency operations within 72 h for one or more of the following indications: (1) hemodynamic instability, (2) worsening metabolic acidosis, (3) severe progressive cyanosis, or (4) escalating intensive care support as described in other studies [6, 7]. All the 54 patients received intubation and mechanical ventilation pre-op, we also performed conventional hyperventilation and pharmacological interventions, such as treatment with milrinone or dopamine, with the aim of controlling PH, maintaining stable hemodynamics and restoring homeostasis [5, 8]. Forty-four of these patients received emergency operations between 24 to 72 h after their hemodynamics and internal milieu were stabilized, 32 patients received vasoactive medications (milrinone), these patients were referred to as stable group (SG). Additionally, 10 patients received emergency operations within 24 h after diagnosis due to uncorrected acidosis or progressive cardiovascular collapse, and 8 patients received vasoactive medications (3 patients received milrinone,5 patients received milrinone and dopamine), we refer to these patients as the unstable group (UG).

Echocardiography combined with computed tomography angiography (CTA) was performed in all patients in the SG group. CTA is an accurate, noninvasive, economical diagnostic modality for the preoperative evaluation of TAPVC [3, 9, 10]. Echocardiography was performed in all of the patients in the UG group, and 7 patients underwent CTA. None of the patients in either group underwent cardiac catheterization, given the invasiveness of the procedure. PVO was defined if Doppler examination revealed a pulmonary venous flow velocity > 2 m/s [3, 11, 12]. Hospital mortality was defined as mortality within 30 days of surgery or before discharge. All other deaths were considered late mortality [3, 13]. Arrhythmia was defined sustained arrhythmia that requires medicine such as supraventricular tachycardia and frequent ventricular premature. Low cardiac output was defined as clinical signs or symptoms (eg, tachycardia, oliguria, poor perfusion, or cardiac arrest) with or without a widened arterial-mixed venous oxygen saturation difference or metabolic acidosis. Brain injury from cardiac surgery was defined as a spectrum of disorders, including stroke, encephalopathy, and cognitive dysfunction, even abnormal in the electroencephalo-graph (EEG).

### Long-term follow-up

All surgical patients who were discharged alive from the hospital were required to return for outpatient follow-up visits after the initial operation at the first, third and sixth, twelfth months postoperation and annually thereafter. Some patients were required to visit local hospitals for routine examinations. Any abnormal examination result or suspicious change in the cardiac condition prompted a return visit for further evaluation. During the follow-up period, echocardiography was performed routinely.

### Surgical technique

The surgery was performed using standard cardiopulmonary bypass (CPB). Ice slush was used for myocardial protection in all patients. HTK cardioplegia was used routinely in both group. The type of CPB circuit is depend on the weight, In our hospital, the 3/16 in. of the arterial tube diameter was chosen. The priming strategy is synthetic colloid, Blood-based priming solutions, albumin, Lactated Ringer’s. A temporal reduction in bypass flow could be used to achieve satisfactory intraoperative visualization if needed. The operation was performed through a median sternotomy, there was minimal manipulation of the heart until CPB was established, and the ductus arteriosus was dissected and ligated following the bypass.

In patients with supracardiac TAPVC, a long incision was made at the pulmonary venous confluence, and then an incision was made at the corresponding position on the left atrium. Direct side-to-side anastomosis was performed between these two chambers from outside with a running suture to ensure precise geometric alignment and avoid tension, torsion, and rotation; the anastomosis was made to be as large as possible [14]. This approach was described by Tucker [15]. The atrial septal defect (ASD) was directly closed with a patch. In patients with cardiac TAPVC, the coronary sinus was unroofed, and a wide tunnel was created between the left atrium and the coronary sinus [16]. In patients with infracardiac TAPVC, the heart was lifted, and a wide anastomosis was generated between the pulmonary venous chamber and the left atrium with the same procedure as that used for the supracardiac form [14].

### Statistical analysis

Quantitative data are expressed as the mean ± standard deviation as appropriate. All *P* values were two-sided, and *P* < 0.05 was considered statistically significant. Age, sex, and type were determined by using the chi-square test. Quantitative data (weight, bypass time, and clamp time) were analyzed by using a t test. Survival data are presented by means of the Kaplan-Meier method.

## Results

There were 44 patients in the SG group, of whom 31 were male and 13 were female. 3 patients were premature, the rest was term infant, PDA was the most common comorbidities (28 patients), the next is tricuspid valve regurgitation (21 patients), and 9 patients were prenatally diagnosed. The median weight at surgery was 4.3 ± 1.4 kg (1.7–5.5 kg), and the median age at surgery was 72.4 ± 85.4 days (1–455 days), the preoperative lowest pH was 7.22 ± 0.11, the preoperative highest plasma lactate was 2.21 ± 2.56 mmol/L, the preoperative Aspartate transaminase (AST) was 78.09 ± 10.84 U/L, and the preoperative peak creatinine and preoperative peak urea were 40.37 ± 16.89 μmol/L and 4.29 ± 2.47 mg/dl respectively, the gestational age was 38.3 ± 2.31 weeks. PDA was the most common comorbidities (28 patients), the next is tricuspid valve regurgitation (21 patients). One patient was 15 months old and lived in a remote mountain area. He had respiratory and hemodynamic instability that required mechanical ventilation. Twenty patients were diagnosed with prerepair PVO; of these patients, 13 exhibited the supracardiac type, 6 exhibited the cardiac type, and 1 exhibited the mixed cardiac type, and the flow of PVO was 2.52 ± 0.24 m/s. There were 10 patients in the UG group, of whom 6 were male and 4 were female, only 1 patient was premature, the rest were term infant. PDA (6 patients) and severe tricuspid valve regurgitation (5 patients) was the top two comorbiditie, and 3 patients were prenatally diagnosed. The median weight at surgery was 4.3 ± 0.9 kg (2.8–4.9 kg), and the median age at surgery was 37.5 ± 25.5 days (6–90 days). The preoperative lowest pH was 7.15 ± 0.13, the preoperative highest plasma lactate was 1.77 ± 0.80 mmol/L, the preoperative AST was 90.43 ± 100.64 U/L, and the preoperative peak creatinine and preoperative peak urea were 33.31 ± 10.48 μmol/L and 3.59 ± 2.37 mg/dl respectively, the gestational age was 39.40 ± 1.26 weeks. Preoperative PVO occurred in 6 patients; of these patients, 2 exhibited the supracardiac type, 1 exhibited the cardiac type, and 3 exhibited the infracardiac type, and the flow of PVO was 2.45 ± 0.23 m/s. Patient demographics are listed in Table 1. The comparison between the variables were without significance.

**Table 1:** Demographics and clinical characteristics of the study cohort

Demographics		SG (*n* = 44)	UG (*n* = 10)	t/χ^2^	*p*
Sex	Male	31	6	0.413	0.521
	Female	13	4		
Age at presentation, mean ± SD (d)		72.4 ± 85.4	37.5 ± 25.5	1.273	0.209
Weight at presentation, mean ± SD (kg)		4.3 ± 1.4	4.3 ± 0.9	0.066	0.948
PVO		45.5% (20/44)	60% (6/10)	0.927	0.485
gestational age(w)		38.3 ± 2.31	39.40 ± 1.26	−1.451	0.153
the flow of PVO(m/s)		2.52 ± 0.24	2.45 ± 0.23	0.679	0.504
Preoperative Lowest pH		7.22 ± 0.11	7.15 ± 0.13	1.923	0.06
Preoperative Highest plasma lactate (mmol/L)		2.21 ± 2.56	1.77 ± 0.80	0.533	0.596
Preoperative AST		78.09 ± 10.84	90.43 ± 100.64	−0.33	0.740
Preoperative Peak creatinine (μmol/L)		40.37 ± 16.89	33.31 ± 10.48	1.262	0.213
Preoperative Peak urea (mg/dl)		4.29 ± 2.47	3.59 ± 2.37	0.924	0.360
TAPVC type	Supracardiac	23	3	4.123	0.248
	Cardiac	15	4		
	Infracardiac	4	3		
	Mixed	2	0		

The mean CPB duration was 134.9 ± 42.9 (58–303) minutes in the SG group and 133.0 ± 41.0 (66–193) minutes in the UG group (*p* = 0.901). The mean aortic cross-clamp (ACC) duration was 68.2 ± 25.1 (36–135) minutes in the SG group and 54.7 ± 21.5 (range, 27–93) minutes in the UG group (*p* = 0.123). The median duration of ventilator support was 8.1 ± 4.6 (2–13) days in the SG group and 4.9 ± 2.1 (2–18) days in the UG group (*p* = 0.008). the Post-op days in ICU and Days of hospitalization were 8.64 ± 4.04 days and 19.9 ± 4.27 days in the SG group and 5.6 ± 2.01 days and 14.7 ± 1.75 days (*P* = 0.026 and 0.002). The flow of pulmonary venous anastomosis was 1.18 ± 0.32 m/s in the SG group and 1.26 ± 0.22 m/s in the UG group (*p* = 0.008). Patient demographics are listed in Table 2.

**Table 2:** Surgical data and perioperative data

	SG (*n* = 44)	UG (*n* = 10)	t/χ2	*p*
Mean aortic cross-clamp (ACC) duration (min)	68.2 ± 25.1	54.7 ± 21.5	1.569	0.123
Mean cardiopulmonary bypass duration (min)	134.9 ± 42.9	133.0 ± 41.0	0.125	0.901
Post-op days in ICU(d)	8.64 ± 4.04	5.6 ± 2.01	2.300	0.026
Days of hospitalization(d)	19.9 ± 4.27	14.7 ± 1.75	3.310	0.002
the flow of pulmonary venous anastomosis(m/s)	1.18 ± 0.32	1.26 ± 0.22	−0.725	0.472
Post-op duration of ventilation (d)	8.1 ± 4.6	4.9 ± 2.1	2.892	0.008
Mortality (n/%)	12 (27.3%)	2 (20%)	0.224	0.636

Table 3 shows the comparison of means and χ2 test results for the complications encountered postoperatively in both groups. Delayed sternal closure was performed in 5 patients and 1 patient in the SG group and UG group, respectively (*P* = 0.901). Low cardiac output occurred in 9 patients and 2 patients (*P* = 0.974). Arrhythmia occurred in 6 patients and 1 patient (*P* = 0757). One patient in the SG group and no patient in the UG group developed PVO (*P* = 0.630). Diaphragmatic paralysis was found in 1 patient in the SG group and none in the UG group (*P* = 0.630); peritoneal dialysis was performed in 8 patients in the SG group and 3 patients in the UG group (*P* = 0.402), these patients with no need for peritoneal dialysis in the long term, the renal function was normal in these patients. Brain injury was found in 3 patients in the SG group and 1 patient in the UG group (*P* = 0.571). Blood transfusion was 1.51 ± 0.37 U in the SG group and 1.35 ± 0.36 U in the UG group (*P* = 0.202). Hospital acquired infections was found in 7 patients in the SG group and 3 patients in the UG group (*P* = 0.571). There were no statistically significant differences between the two groups regarding the complications encountered postoperatively.

**Table 3:** Postoperative complications in the two groups

	SG (*n* = 44)	UG (*n* = 10)	t/χ2	*p*
Low cardiac output	9 (20.5%)	2 (20%)	0.001	0.974
Peritoneal dialysis	8 (18.2%)	3 (30%)	0.702	0.402
Arrhythmia	6 (13.6%)	1 (10%)	0.095	0.757
Hospital acquired infections	7	3	1.072	0.370
Blood transfusion(u)	1.51 ± 0.37	1.35 ± 0.36	1.293	0.202
Brain injury	3	1	0.120	0.571
Postoperative PVO	1 (2.3%)	0	0.232	0.630
Diaphragmatic paralysis	1 (2.3%)	0	0.232	0.630
Delayed sternal closure	5 (11.4%)	1 (10%)	0.015	0.901

There were 12 (27.3%) and 2 hospital mortalities (20%) in the SG group and UG group, respectively, but the difference was not significant (*p* = 0.636). Among the 14 patients who died within 30 days of surgery, the main causes of death were as follows: low output failure (*n* = 8), pulmonary hypertensive crisis (*n* = 3), multiple organ dysfunction syndrome (*n* = 1) and failure to wean from ventilation (*n* = 1). All patients were weaned off CPB with inotropic support, except for 1 patient in the SG group.

**Fig. 1:**
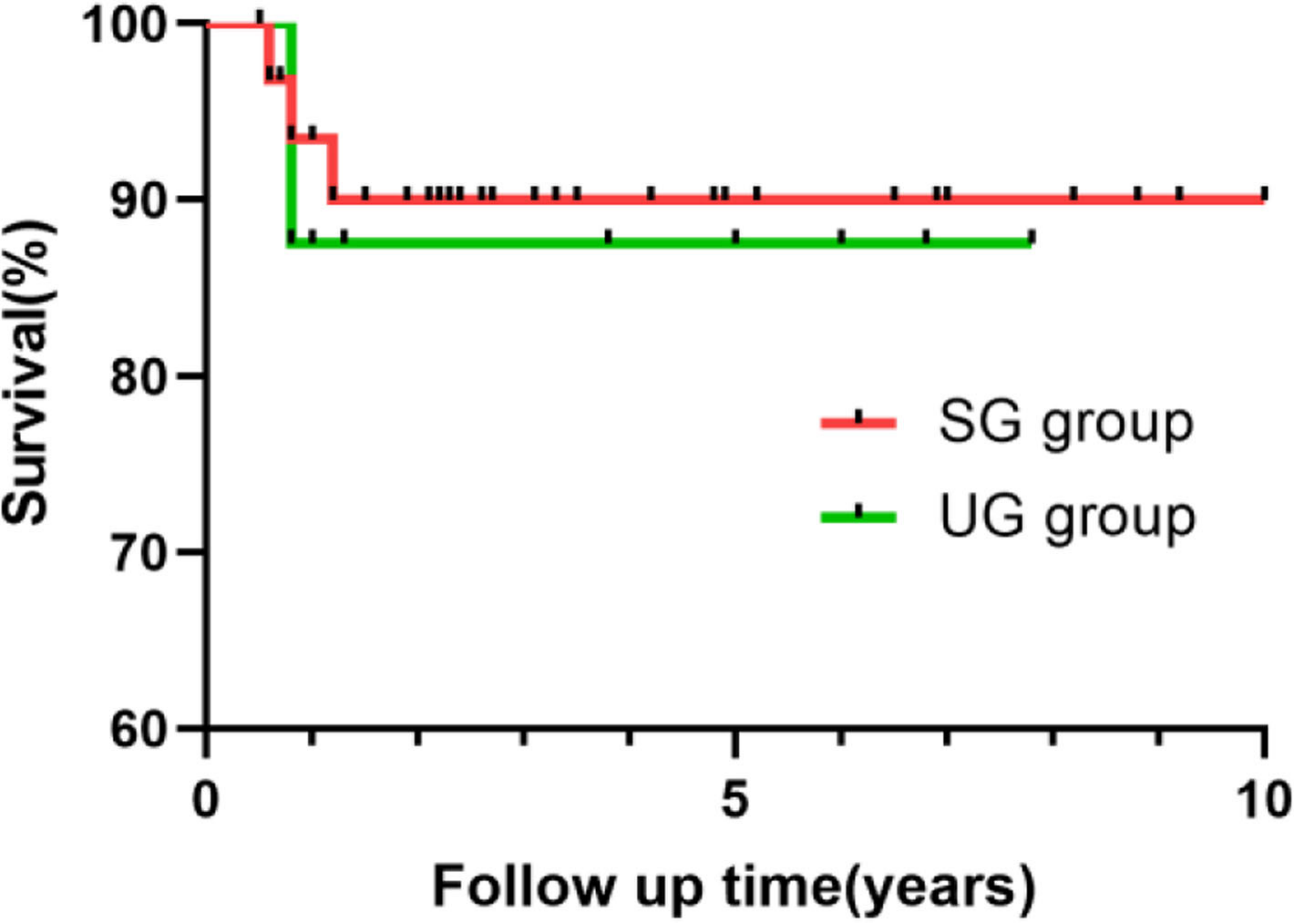
Actuarial survival at 5 years in the two groups

The median follow-up time for survivors was 5.9 years (0.4 to 9.2 years). In the SG group, 2 patients were lost to follow-up. Three late deaths occurred after the operation, and all occurred during follow-up at 7 months, 9 months and 1.2 years due to PVO. The survival at 5 years was 89.9% in the SG group (Fig. 1). In the UG group, there was one late mortality of a patient with infracardiac TAPVC 9 months after the operation because of PVO. The survival at 5 years was 87.5% (Fig. 1). There was no difference in survival between the two groups at the latest follow-up (SG group 89.9% versus UG group 87.5%, *p* = 0.8115).

## Discussion

TAPVC is a frequently encountered problem in some heart centers. Some patients have PVO or a delayed diagnosis, and the condition can suddenly worsen, requiring mechanical ventilation, volume substitution, and inotropic support. Briefly, an emergency operation should be performed in such patients. In this case, someone would make efforts to obtain stable hemodynamics and maintain a stable internal milieu, and then, an emergency operation would be performed. In other cases, an emergency operation would be performed immediately, medical efforts are ineffective at managing the hemodynamic and metabolic problems that subsequently occur. Mechanical ventilation, volume substitution and inotropic support do little to help maintain stable hemodynamics and a stable internal milieu. A megadose of vasoactive medications can even worsen cardiac function. Previous studies concluded that immediate emergency surgery was the best strategy, regardless of the high operative risk [5, 17, 18].

In contrast, stabilizing the patient’s condition before surgery is considered important for improving the surgical outcome. Some studies have reported the use of vasoactive medications such as dopamine and milrinone to stabilize hemodynamics and correct metabolic acidosis, and when the patient’s condition was stable, an operation was performed. Although one study reported a reduction in mortality, there was insufficient evidence to support this conclusion [17]. In addition, other palliative surgical techniques, such as balloon dilatation, stenting and atrial septectomy, are alternative approaches to relieve cyanosis and improve the patient’s clinical state. These palliative techniques have been shown to be somewhat successful for decompressing pulmonary venous pressure with restricted ASD or PVO. These techniques are associated with some inherent complications, such as the need for reintervention (due to occlusion of the stent or the recurrence of PVO) and the need for anticoagulation. Research has shown that the mortality rate is 38% at 1 year and that the reintervention rate is 58% at 1 year [2, 5, 19]. Moreover, not all patients could tolerate these palliative measures, and patients needed to undergo surgery to correct the anomalous pulmonary veins [5]. Extracorporeal membrane oxygenation (ECMO) is also an alternative approach, but its cost and serious complications restrict its popularization [2, 20].

A finding of our review is that the mortality rate was higher in the SG group, though without significance. The reasons might be as follows. First, according to some clinical reports, there is evidence that the long-term application of cardiovascular drugs does not improve myocardial function or survival [21–23]. Indeed, some researchers have shown that the use of these drugs results in myocardial injury, the destruction of myocardial function via myocardial cellular apoptosis and a decrease in heart contractility, which substantially affects myocardial function [21]. Second, due to severe dyspnea and aggravated metabolic acidosis, these patients’ hearts contracted weakly, and their oxygenation continued to worsen unless the malformation was corrected. Additionally, their cardiac function worsened during the preoperative preparation period [1].

Another more important and reasonable finding is that the duration of ventilation and days of hospitalization was significantly longer in the SG group. Longer postoperative mechanical ventilation results in a higher risk of ventilator-associated pneumonia and prolongs the length of stay. Additionally, studies have shown that prolonged postoperative mechanical ventilation is the most significant predictor of early mortality [24]. Long postoperative mechanical ventilation is usually attributed to the presence of chest infection or a worsened condition of heart failure. Therefore, the longer postoperative mechanical ventilation period in the SG group might be explained as follows. First, an infusion of excess fluid, aiming at stabilizing hemodynamics and correcting metabolic acidosis before the operation, has been demonstrated to worsen mechanical ventilation-related complications and aggravate pneumonia in critically ill patients [6, 24]. Second, an infusion of excess fluid and long-term application of cardiovascular drugs can also weaken myocardial functioning and prolong mechanical ventilation, as discussed above.

Our report indicates that early mortality among these critically ill patients (in both the SG group and the UG group) is higher than the overall TAPVC mortality reported by others. It seems that those who undergo emergency operations have a higher overall mortality rate [2]. Clearly, patients with respiratory distress and metabolic acidosis or PVO are the most severely ill patients, and these factors have been shown to be risk factors for mortality in many studies. The urgency of surgery itself is also a risk factor for mortality [1], and therefore, such patients are expected to have a higher mortality rate [1, 4, 11]. Moreover, in some patients, the hemodynamic status was still unstable after all of the measures mentioned above had been implemented, and an emergency operation became the only choice when the palliative techniques were abandoned, considering the aforementioned defects. Regarding the emergency operations themselves, the early mortality is similar to the result of other reports [1, 3, 24].

Postoperative complications, such as low cardiac output and arrhythmia, were not significantly different between the two groups. Long-term survival also did not differ between the two groups. Survivors in the two groups (among critically ill patients) appear to have similar long-term outcomes, Zhao K et al. also reported a similar result [1]. Postoperative patients may have late pulmonary vein obstruction, While patients undergoing an emergency operation are unlikely to be more predisposed to stenosis. The statistical analyses in our series showed a good outcome, with freedom from medication and normal activity.

There are multiple limitations of this study. This was a retrospective single-center study, and the sample size was small; thus, the results may not be applicable to the population as a whole. TAPVC often combined with various malformations. Although cases combined with malformations of high risk for death were excluded, other concurrent abnormalities could still affect survival outcomes.

## Conclusion

Taken together, our experience with these critically ill patients is as follows. Given the lower mortality rate (though without significance compared to the SG group), shorter duration of ventilation and days of hospitalization in the UG group, emergency operations should be performed without any delay, it can reduce duration of mechanical ventilation and Days of hospitalization without reducing mortality.

## Data Availability

The data and materials in the manuscript are available, and the original data for the relevant results are owned by myself; I can be contacted if needed.

## Abbreviations

TAPVC: Total anomalous pulmonary venous connection
PVO: Pulmonary venous obstruction
SEO: Subemergency operation
EO: Emergency operation
CPB: Cardiopulmonary bypass
ACC: Duration and mean aortic cross-clamp
PH: Pulmonary hypertension
PVO: Because of pulmonary venous obstruction
CTA: Computed tomography angiography
ASD: Atrial septal defect
ECMO: Extracorporeal membrane oxygenation
AST: Aspartate transaminase

## References

[1] Zhao K, Wang H, Wang Z, Zhu H, Fang M, Zhu X, Zhang N, Song H (2015). Early-and intermediate-term results of surgical correction in 122 patients with total anomalous pulmonary venous connection and biventricular physiology. J Cardiothorac Surg.

[2] Choi EY, Lee CH, Park SJ, Jang SI, Kim ES (2019). Assessing the recently noted surgical outcome of isolated total anomalous pulmonary venous connection repair: a single-secondary center experience. J Card Surg.

[3] Shi G, Zhu Z, Chen J, Ou Y, Hong H, Nie Z, Zhang H, Liu X, Zheng J, Sun Q (2017). Total anomalous pulmonary venous connection: the current management strategies in a pediatric cohort of 768 patients. Circulation..

[4] Karamlou T, Gurofsky R, Al Sukhni E, Coles JG, Williams WG, Caldarone CA, Van Arsdell GS, McCrindle BW (2007). Factors associated with mortality and reoperation in 377 children with total anomalous pulmonary venous connection. Circulation..

[5] Sarmast H, Takriti A (2019). A new palliative surgical technique for high risk Total anomalous pulmonary venous connection (Sarmast-Takriti shunt). J Cardiothorac Surg.

[6] Harada, Takeaki, Nakano, Toshihide, Oda, Shinichiro, Kado and Hideaki. Surgical results of total anomalous pulmonary venous connection repair in 256 patients. Interact Cardiovasc Thorac Surg. 2019. 10.1093/icvts/ivy267 30202975

[7] Pruetz JD, Carroll C, Trento LU, Chang RK, Detterich J, Miller DA, Sklansky M (2014). Outcomes of critical congenital heart disease requiring emergent neonatal cardiac intervention. Prenat Diagn.

[8] Kiziltepe U, Eyileten ZB, Uysalel A, Akalin H (2003). Acute pulmonary hypertensive crisis after TAPVC repair treated with atrial septectomy with inflow occlusion. Int J Cardiol.

[9] Xiang Y, Cheng G, Jin K, Zhang X, Yang Y (2018). Computed tomography findings and preoperative risk factors for mortality of total anomalous pulmonary venous connection. Int J Card Imaging.

[10] Husain SA, Maldonado E, Rasch D, Michalek J, Taylor R, Curzon C, Neish S, Calhoon JH (2012). Total anomalous pulmonary venous connection: factors associated with mortality and recurrent pulmonary venous obstruction. Ann Thorac Surg.

[11] Ricci M, Elliott M, Cohen G, Catalan G, Stark J, De Leval M, Tsang V (2003). Management of pulmonary venous obstruction after correction of TAPVC: risk factors for adverse outcome. Eur J Cardiothorac Surg.

[12] Kirshbom PM, Myung RJ, Gaynor JW, Ittenbach RF, Paridon SM, DeCampli WM, Karl TR, Spray TL (2002). Preoperative pulmonary venous obstruction affects long-term outcome for survivors of total anomalous pulmonary venous connection repair. Ann Thorac Surg.

[13] Yong MS, Yaftian N, Weintraub RG, Brizard CP, d'Udekem Y, Konstantinov IE (2017). Outcomes of surgery for mixed total anomalous pulmonary venous drainage in children. Semin Thorac Cardiovasc Surg.

[14] Chowdhury UK, Airan B, Malhotra A, Bisoi AK, Saxena A, Kothari SS, Kalaivani M, Venugopal P (2008). Mixed total anomalous pulmonary venous connection: anatomic variations, surgical approach, techniques, and results. J Thorac Cardiovasc Surg.

[15] Tucker BL, Lindesmith GG, Stiles QR, Meyer BW (1976). The superior approach for correction of the supracardiac type of total anomalous pulmonary venous return. Ann Thorac Surg.

[16] Malm J. Secundum atrial septal defects and associated anomalous pulmonary venous drainage. Craft Surg. 1964:546–62.

[17] van Son JA, Hambsch J, Kinzel P, Haas GS, Mohr FW (2000). Urgency of operation in infracardiac total anomalous pulmonary venous connection. Ann Thorac Surg.

[18] Yun T-J, Al-Radi OO, Adatia I, Caldarone CA, Coles JG, Williams WG, Smallhorn J, Van Arsdell GS (2006). Contemporary management of right atrial isomerism: effect of evolving therapeutic strategies. J Thorac Cardiovasc Surg.

[19] Gao X, Nie Z, Yanqiu O, Biaochuan H, Yuan H, Yanji Q, Liu X (2017). Comparison between two surgical techniques to repair total anomalous pulmonary venous connection using propensity scoreanalysis. J Sun Yat-sen Univ Med Sci.

[20] Raisher BD, Grant JW, Martin TC, Strauss AW, Spray TL (1992). Complete repair of total anomalous pulmonary venous connection in infancy. J Thorac Cardiovasc Surg.

[21] Hoffman TM, Wernovsky G, Atz AM, Kulik TJ, Nelson DP, Chang AC, Bailey JM, Akbary A, Kocsis JF, Kaczmarek R, Spray TL, Wessel DL (2003). Efficacy and safety of milrinone in preventing low cardiac output syndrome in infants and children after corrective surgery for congenital heart disease. Circulation..

[22] Kruger AD, Mundt A, Oldag D, Hinsenbrock KP (1985). High-dosage adrenaline therapy in low cardiac output syndrome following aortic valve replacement. Anaesthesiol Reanim.

[23] Coffin LH, Ankeney JL, Beheler EM (1966). Experimental study and clinical use of epinephrine for treatment of low cardiac output syndrome. Circulation..

[24] Elamry E, Alkady HM, Menaissy Y, Abdalla O (2019). Predictors of in-hospital mortality in isolated total anomalous pulmonary venous connection. Heart Surg Forum.

